# Physiological and Transcriptional Responses to Saline Irrigation of Young ‘Tempranillo’ Vines Grafted Onto Different Rootstocks

**DOI:** 10.3389/fpls.2022.866053

**Published:** 2022-06-06

**Authors:** Ignacio Buesa, Juan G. Pérez-Pérez, Fernando Visconti, Rebeka Strah, Diego S. Intrigliolo, Luis Bonet, Kristina Gruden, Maruša Pompe-Novak, Jose M. de Paz

**Affiliations:** ^1^Instituto Valenciano de Investigaciones Agrarias, Centro para el Desarrollo de la Agricultura Sostenible, Unidad Asociada al CSIC “Riego en la Agricultura Mediterránea”, Valencia, Spain; ^2^Ecophysiologie et Génomique Fonctionnelle de la Vigne, Institut National de la Recherche Agronomique, Institut des Sciences de la Vigne et du Vin, Villenave d’Ornon, France; ^3^Research Group on Plant Biology Under Mediterranean Conditions, Department of Biology, University of the Balearic Islands, Palma, Spain; ^4^Centro de Investigaciones sobre Desertificación, Departmento de Ecología (CSIC, UV, GV), Valencia, Spain; ^5^Department of Biotechnology and Systems Biology, National Institute of Biology, Ljubljana, Slovenia; ^6^Jožef Stefan International Postgraduate School Ljubljana, Ljubljana, Slovenia; ^7^School for Viticulture and Enology, University of Nova Gorica, Vipava, Slovenia

**Keywords:** osmotic adjustment, gas exchange, gene expression, water relations, *Vitis vinifera* L. (grapevine), salinity tolerance

## Abstract

The use of more salt stress-tolerant vine rootstocks can be a sustainable strategy for adapting traditional grapevine cultivars to future conditions. However, how the new M1 and M4 rootstocks perform against salinity compared to conventional ones, such as the 1103-Paulsen, had not been previously assessed under real field conditions. Therefore, a field trial was carried out in a young ‘Tempranillo’ (*Vitis vinifera* L.) vineyard grafted onto all three rootstocks under a semi-arid and hot-summer Mediterranean climate. The vines were irrigated with two kinds of water: a non-saline Control with EC of 0.8 dS m^–1^ and a Saline treatment with 3.5 dS m^–1^. Then, various physiological parameters were assessed in the scion, and, additionally, gene expression was studied by high throughput sequencing in leaf and berry tissues. Plant water relations evidenced the osmotic effect of water quality, but not that of the rootstock. Accordingly, leaf-level gas exchange rates were also reduced in all three rootstocks, with M1 inducing significantly lower net photosynthesis rates than 1103-Paulsen. Nevertheless, the expression of groups of genes involved in photosynthesis and amino acid metabolism pathways were not significantly and differentially expressed. The irrigation with saline water significantly increased leaf chloride contents in the scion onto the M-rootstocks, but not onto the 1103P. The limitation for leaf Cl^–^ and Na^+^ accumulation on the scion was conferred by rootstock. Few processes were differentially regulated in the scion in response to the saline treatment, mainly, in the groups of genes involved in the flavonoids and phenylpropanoids metabolic pathways. However, these transcriptomic effects were not fully reflected in grape phenolic ripeness, with M4 being the only one that did not cause reductions in these compounds in response to salinity, and 1103-Paulsen having the highest overall concentrations. These results suggest that all three rootstocks confer short-term salinity tolerance to the scion. The lower transcriptomic changes and the lower accumulation of potentially phytotoxic ions in the scion grafted onto 1103-Paulsen compared to M-rootstocks point to the former being able to maintain this physiological response in the longer term. Further agronomic trials should be conducted to confirm these effects on vine physiology and transcriptomics in mature vineyards.

## Introduction

Changes in the Mediterranean and related semi-arid climates are expected shortly, leading to temperature increases and more frequent and longer drought periods (Döll, 2002). These will increase crop water demand, while simultaneously reducing the availability of quality water (Schultz, 2017). Since in most grapevine-growing regions, freshwater is a scarce resource (Medrano et al., 2015), the use of alternative waters, such as wastewaters often high in salts, will be more and more needed to mitigate drought stress (Mirás-Avalos and Intrigliolo, 2017). Besides, conventional waters, such as underground water, can indeed be of low quality due to excessive concentrations of soluble salts (Cl^–^ and/or Na^+^), with an electrical conductivity over 3 dS m^–1^ (Pérez-Pérez et al., 2015). This lack of water quality poses a challenge to the sustainability of deficit irrigation in viticulture, as this irrigation strategy could aggravate the effects of salinity (van Leeuwen et al., 2019).

Excessive soil salinity can cause water loss, nutrient deficiency, oxidative stress, photoinhibition, growth inhibition, and induce many metabolic and transcriptomic changes leading to physiological damage (Walker et al., 1997; Kumari et al., 2015; Saha et al., 2015; Upadhyay et al., 2018; Zhou-Tsang et al., 2021). Previous studies have demonstrated that among plant responses to salinity, mechanisms that control ion uptake, transport, and balance, as well as hydric regulation, photosynthesis, cell division, osmotic adjustment, enzymatic activities, antioxidant production, stress signaling, and regulation of root barriers play critical roles in plant tolerance to salinity (Gong et al., 2011; Shahid et al., 2020; Zhou-Tsang et al., 2021).

The *Vitis vinifera* L. is a crop classified as moderately sensitive to salinity (Maas and Hoffman, 1977; Cramer et al., 2007), with a soil saturation extract electrical conductivity at 25°C yield threshold (EC_t_) of 2.6 dS m^–1^ (Walker et al., 2002). The tolerance of grapevines to salinity depends on multiple factors and, particularly, on plant genetics, soil and climate characteristics, and the rate and length of the stress, to which vines are subjected (Maas and Hoffman, 1977; Zhang et al., 2002; Cramer et al., 2007; Chaves et al., 2009; Mirás-Avalos and Intrigliolo, 2017). Understanding the physiological and transcriptomic responses of grapevine to saline water is essential to prevent and mitigate potential negative effects on vine performance and grape composition (Ollat et al., 2016). Moreover, the contradictory effects of irrigation with saline or wastewater on vine performance and grape composition (Walker et al., 2004, 2007; Stevens et al., 2011; Mirás-Avalos and Intrigliolo, 2017) point toward the existence of important knowledge gaps regarding the effects of salinity and the salt tolerance mechanisms in *Vitis* spp. (Zhou-Tsang et al., 2021). Microarray studies of pot-grown own-rooted vines of CVS ‘Cabernet Sauvignon,’ ‘Razegui,’ and ‘Shiraz’ revealed that salinity stress impaired photosynthesis and increased the expression of some transcription factors and genes related to ROS scavenging, abscisic acid, and osmoprotectants such as various sugars and proline (Cramer et al., 2007; Daldoul et al., 2010). High throughput sequencing studies of potted cv. ‘Thompson Seedless’ and cv. ‘Summer Black’ under greenhouse conditions implicated the activity of genes involved in cell wall modulation, various cation and ABC transporters, signal transduction genes, HSPs, and biotic stress-related genes (Guan et al., 2018; Das and Majumder, 2019).

The ‘Tempranillo’ cultivar has been specifically classified as moderately salt-sensitive as well, showing growth decreases attributable to osmotic effects rather than to ion-specific toxicities (Urdanoz and Aragüés, 2009). Nonetheless, since grapevine yield potential under saline conditions is related to the root-zone salinity, the plant portion that primarily deals with soil salinity is not the scion, but the rootstock. Among the characteristics of the different rootstock that contribute to enhancing grapevine tolerance to salinity, there is its ability to exclude and not transport salt to the shoots; besides, there is also the vigor it confers to the scion (Walker et al., 2002, 2014; Munns et al., 2020). Additionally, rootstock can have a great influence on stomatal regulation in response to water and salinity stress, even more than the scion itself (Lavoie-Lamoureux et al., 2017). For instance, rootstock can affect the osmotic adjustment response, which is one of the main physiological processes, whereby the vine responds to salinity (Keller, 2010; Haider et al., 2019). This consists of the active accumulation of solutes, thus increasing leaf relative water content and turgor (Barrios-Masias et al., 2018). Regarding this, several studies are reporting that the rootstocks with lower osmotic adjustment capacity are those with greater capacity to restrict the leaf accumulation of Na^+^ and Cl^–^, thus, preventing their possible phytotoxic effects (Stevens and Walker, 2002; Zhang et al., 2002), and minimizing their accumulation in the grape juice and wine in the long-term (Walker et al., 2004, 2014; Teakle and Tyerman, 2010).

American *Vitis* species, especially *V. rupestris*, *V. riparia*, and *V. berlandieri* are tolerant of saline and limestone soils (Williams et al., 1994; Ferlito et al., 2020). Some rootstocks derived from these species such as Ramsey (*V. champini*), 1103 Paulsen (1103P), 110 Richter, 140 Ruggeri, and 101–14 Mgt can exclude much salt (chiefly Na^+^ and Cl^–^) from root uptake and root-to-shoot transport (Walker et al., 2004, 2010; Gong et al., 2011). For instance, some of the most salinity-tolerant rootstocks, such as 140 Ruggeri and 1103 Paulsen, have an EC_t_ value of up to 3.3 dS m^–1^ (Walker et al., 2002; Zhang et al., 2002; Tregeagle et al., 2006). Conversely, rootstocks, such as SO4 and 3309C, are characterized by being very sensitive to salinity with an EC_t_ value below 1.8 dS m^–1^ (Walker et al., 2010). Given the relatively narrow genetic pool within the commercial grapevine rootstocks and the significant genetic diversity of the genus *Vitis*, identifying salinity-tolerant grapevine rootstocks is a great opportunity to enhance viticulture sustainability (Schultz and Stoll, 2010). For instance, differential gene expression has been observed in potted *Vitis vinifera L.* ssp. *sylvestris* with different short-term salinity tolerance in greenhouse conditions (Askri et al., 2012). Therefore, a better understanding of the rootstock physiological, metabolomic, and transcriptomic mechanisms underlining salt stress tolerance is essential to improve breeding programs aimed at adapting to climate change (Ollat et al., 2016). In this sense, new information about salinity tolerance conferred by rootstocks is needed (Keller, 2010; Marín et al., 2021). Grapevine rootstock breeding programs, such as the one carried out by the University of Milan (Italy) with the M-series, are very promising for coping with water salinity (Meggio et al., 2014) and can benefit a lot from the results of field trials.

Therefore, the objective of the present research was to evaluate the physiology and transcriptomics underlying the performance against salinity of two new rootstocks, M1 and M4, compared to the well-known salinity-tolerant 1103P (Walker et al., 2010; Bianchi et al., 2020). In this work the experimental hypothesis was that the M-rootstocks may confer better salinity tolerance to the scion than the 1103P through enhanced uptake of salt-stress-contesting ions such as calcium, as well as vigor declining ability, in the case of the M1 (Porro et al., 2013; Vannozzi et al., 2017), and because of the leaf build-up of inorganic osmolytes and sodium-antagonists, such as potassium, in the case of the M4 (Meggio et al., 2014). In comparison to the M-rootstocks, the 1103P stands out for its ability to exclude Cl^–^ from uptake. Aiming at mimicking commercial conditions, the experiment was performed under field conditions and tried to isolate the salinity effect by fully irrigating the vines. Although the vineyard was under establishment, to our best knowledge, these grapevine rootstocks had not been previously tested against salinity under conditions so close to real practice. Besides, in contrast to previous comparative studies between these grapevine rootstocks in this work, all determinations were carried out directly in the scion. This was done considering that the scion is an integrator of rootstock-induced effects (Gambetta et al., 2012; Cookson et al., 2013). Finally, by assessing a young vineyard, i.e., one with a non-extensive root system, the physiological response to salinity could be studied ensuring that most of the roots were effectively under the intended salinity.

## Materials and Methods

### Vineyard Site and Experimental Design

The experiment was undertaken in 2019 in a ‘Tempranillo’ (*Vitis vinifera* L.) vineyard located at the IVIA’s experimental station in Moncada, Valencia, Spain (39° 35′ 12′′ N, 0° 24′ 1′′ W, and 55 m.a.s.l). In 2017, the vines were grafted onto three rootstocks in a nursery. The rootstocks were the M1 clone 1 (106/8 × *V. berlandieri*), the M4 clone 1 (41B x *V. berlandieri*) and the 1103 Paulsen clone VCR119 (*V. berlandieri* cv. ‘Resseguier’ nr. 2 × *V. rupestris* cv. ‘Du Lot’) (Marín et al., 2021). Vines were planted in 2018 at a spacing of 0.88 × 2.50 m and guided by a vertical trellis system in a simple “guyot” cordon. As it was a vineyard under establishment, it was decided to constrain the crop load to four clusters per vine to avoid overcropping. Thus, the experimental vines had an average yield of 1.75 kg, i.e., 7.9 t/ha. There were no differences in initial shoot fruitfulness or yield at harvest among treatments.

The climate in the experimental trial was hot-summer Mediterranean (Csa) according to Köppen–Geiger (Rodríguez-Ballesteros, 2016), and semi-arid according to Thornthwaite (De Paz et al., 2004), with an average annual rainfall of 392 mm and reference evapotranspiration (ET_o_) of 1,137 mm. The soil was classified as a Petrocalcic Calcixerept according to the Soil Taxonomy (Soil Survey Staff, 2006) with the petrocalcic horizon constraining root development lying at 0.4–0.5 m depth, and with loam texture (45% sand, 36% silt, and 19% clay), high calcium carbonate equivalent (40%) and, therefore, medium-to-high active calcium carbonate equivalent (6–10%), very low organic matter content (1%), and slight-to-moderate compaction (1.56 ± 0.13 Mg/m^3^ of bulk density).

The vineyard was drip irrigated at 100% of crop evapotranspiration (ET_c_), based on the crop coefficients reported for ‘Tempranillo’ vines by López-Urrea et al. (2012), and the ET_o_ calculated with the Penman–Monteith equation (Allen et al., 1998). Weather conditions were recorded at an automated agro-meteorological station 400 m away from the plot. Importantly, no leaching fraction was adopted. Irrigation was applied through 2 L h^–1^ pressure-compensated emitters spaced at 0.88 m along a single drip line and it began 50 days after budburst, i.e., the day of the year (DOY) 133. This time was selected because then, was when midday Ψ_stem_ values reached –0.8 MPa. As a result, the vine water requirements were met by irrigation events 2-to-3 h long 3-to-5 days a week. Mineral nutrients were provided along the season by fertigation up to the cumulated rates of 30, 20, and 60 kg ha^–1^ of, respectively, N, P_2_O_5_, and K_2_O.

Two irrigation waters were generated by dissolving adequate amounts of reagent grade calcium and sodium chlorides in partially desalinated water. Each irrigation water featured a different electrical conductivity at 25°C (EC_25_), but a common sodium-adsorption ratio (SAR) of 5–7 (mmol L^–1^)^1/2^. This way a sodification effect was avoided, which would have shown up as differences in soil structural stability and nutrient availability between the control and saline water, thus, interfering with the salinity treatment. The control water featured an EC_25_ of 0.8 dS m^–1^ with 2.7, 0.3, and 3.3 mmol L^–1^ of, respectively, Na^+^, Ca^2+^, and Cl^–^, whereas the Saline water featured an EC_25_ of 3.5 dS m^–1^ with 12.7, 6.5, and 25.7 mmol L^–1^ of, respectively, Na^+^, Ca^2+^, and Cl^–^. During the experiment, the soil on the alleyways was tilled and spontaneous weeds in the vine row were controlled by glyphosate herbicide applications.

The experiment followed a complete factorial design to assess the performance of the three rootstocks under the two water quality levels (control and salinity). All treatments, i.e., each combination of rootstock and water quality, had three replicates, thus, resulting in 18 subplots of 10 vines each. The subplots were randomly distributed throughout the vineyard. For the determination of water relations and the measurement of gas exchange parameters, as well as for the transcriptomics, the experimental unit (biological replicate) was the 8th vine of each subplot. For the determination of the leaf nutritional status, leaf area index, and grape quality, the experimental unit consisted of the 8 vines from the 2nd to the 9th in each subplot, thus, leaving the 1st and 10th as guards.

### Field Measurements and Laboratory Determinations

All field measurements and samplings were performed after more than 100 days since the treatments had begun (after 259 ± 2 mm of cumulated irrigation was applied). Specifically, the vine water relations, the gas exchange measurements, and the leaf and berry samplings were performed, on DOY 233. According to the phenological growth stages in the BBCH-scale (Lorenz et al., 1995), the vines on DOY 233 were at stage code 89, which means berries are ripe for harvesting. Total leaf area determinations and harvest were performed, respectively, on DOY 234 and 237. Each laboratory sample was analyzed in duplicate.

Vine water relations were determined in each biological replicate using a pressure chamber (Model 600, PMS Instruments Company, Albany, OR, United States) at pre-dawn (Ψ_pre–dawn_) and midday. At midday, both well-exposed-to-sunlight adult leaves (Ψ_leaf_) and bag-covered leaves (Ψ_stem_) were measured (Santesteban et al., 2019). After the Ψ_leaf_ measurement, this leaf was frozen and stored at –20°C for determination of the leaf osmotic potential (Ψ_π_). Another leaf from the same shoot was collected and re-hydrated for determination of the leaf osmotic potential at full turgor (Ψ_π_^100^). Both Ψ_π_ and Ψ_π_^100^ were measured with a digital osmometer (Wescor, Logan, UT, United States). The leaf turgor potential (Ψ_p_) was calculated as the difference between Ψ_leaf_ and Ψ_π_.

The gas exchange measurements were carried out on two fully exposed and expanded young leaves of each biological replicate using an infrared open gas exchange analyzer system (Li-6400xt, Li-COR, Lincoln, NE, United States). The stomatal conductance (g_s_), net photosynthesis (A_N_), and intrinsic water use efficiency (WUE_i_ = A_N_/g_s_) were measured between 8:00 and 9:30 solar time. The CO_2_ concentration inside the chamber was 400 μmol CO_2_ mol^–1^, and an airflow of 500 μmol min^–1^ was applied. The chamber had an area of 6 cm^2^ exposed to environmental light radiation, with PAR always of 1,500 ± 2 μmol m^–2^ s^–1^. The relative humidity and vapor pressure deficit inside the chamber were 30 ± 2% and 2.25 ± 0.3 kPa.

Leaf nutritional status was determined from samples of 20 fully expanded mature leaves per subplot. Leaves were thoroughly washed with tap water, rinsed with deionized water, and oven-dried at 65°C for 48 h. Next, they were grounded with a disk mill to pass a 200-μm mesh sieve and analyzed for the determination of various macro- and micronutrients. The concentrations of K, Ca, Mg, and Na was determined in the extracts obtained by digestion with HNO_3_:HClO_4_ (2:1) using inductively coupled plasma atomic emission spectrometry (ICP-AES) in an iCAP series 6500 (Thermo Fisher Scientific, Franklin, MA, United States). The total N and C contents were determined by dry combustion with, final N_2_ and CO_2_ measurements (Horneck and Miller, 1998), respectively, using a TruSpec CHNS elemental analyzer (LECO TruSpec Micro Series, St. Joseph, MI, United States). The chloride content was determined in the aqueous extracts obtained by shaking the dried leaf material with deionized water (EC_25_ < 1 μS/cm) for two h by ion chromatography (IC) using an 850 professional IC (Metrohm, Herisau, Switzerland).

The total leaf area per vine was estimated at each biological replicate from allometric relations between shoot length (x, cm) and leaf area per shoot (y, cm^2^) measured with an LI-3100 area meter (LI-COR Biosciences, Lincoln, NE, United States), separating main and lateral shoot (*y* = 17.647 x, *R*^2^ = 0.98*** and *y* = 14.952 x, *R*^2^ = 0.99***, respectively). The leaf area index (LAI) was calculated as the total leaf area per unit of ground surface area.

The berry weight and must composition were determined from 200 randomly-taken berries per subplot. The berries were crushed and hand-pressed through a metal screen filter and the must characteristics, including total soluble solids content (TSS), pH, total titratable acidity (TA), and anthocyanins and polyphenols content, were determined according to reference analysis methods (OIV, 1990).

### Common Data Analyses

Two-way analysis of variance (ANOVA) was used to assess the effects of both factors, rootstock (R) and water quality (WQ), along with its interactions (R × WQ), on the vine water relations, leaf gas exchange, leaf nutrient contents, vine performance, and berry composition. A significant interaction between factors in a two-way ANOVA means that the effects of the factors significantly change in magnitude or direction depending on the levels of the other factor (Snedecor and Cochran, 1989). Therefore, following the two-way ANOVAs, if significant main effects were obtained (*p* < 0.05), but significant interactions between R and WQ were not, the group means were compared using the *post hoc* Duncan test. The ANOVAs and *post hoc* tests were carried out using the Statgraphics Centurion XVI package (version 16.0.07) (Statgraphics Technologies, The Plains, VA, United States). Additionally, regressions were calculated using SigmaPlot (version 11.0) (Systat Software, San Jose, CA, United States).

### RNA Extraction and Sequencing

On DOY 233, immediately after the water relations and gas exchange measurements, one sample of leaves and another one of berries were collected from each biological replicate, thus, making 18 samples in total from each plant organ. Three fully expanded young leaves per plant, from the secondary shoots, and twenty berries were cleaned with a cloth and distilled water before being cut. Leaf samples were wrapped in aluminum foil after removing the petiole. Both leaf and berry samples were immediately frozen in liquid nitrogen at the field. Afterward, samples were stored at –80°C until preparation.

Total RNA was extracted from the samples using an optimized cetyltrimethylammonium bromide (CTAB) method (adapted from Carra et al., 2007), combined with RNA purification on Zymo-Spin Columns (Direct-zol RNA MiniPrep Plus kit, Zymo Research, Irvine, CA, United States). About 50 mg of frozen and powdered plant material was further homogenized with steel beads for 10 min at maximum speed in 800 μL CTAB buffer [Tris-HCl 100 mM, NaCl 2 M, EDTA 25 mM, CTAB 2.0% (w/v), PVP40 2.5% (w/v), and β-mercaptoethanol 2% (v/v), pH = 8] using TissueLyser (Qiagen, Hilden Germany). After the addition of an equal volume of chloroform-isoamyl alcohol 24:1, the sample was vortexed and centrifuged for 10 min at 10,000 *g* and 4°C. The upper aqueous phase was recovered, to which 1.5 volume of pure ethanol was added. After a 30 min precipitation at 4°C, the mixture was transferred into Zymo-Spin Columns. The RNA was further purified according to the manufacturer’s instructions, with an additional washing step and a second prewashing step added to the beginning of the purification process. To elute the RNA, 30 μL of preheated (80°C) DNase/RNase-free water was added to the column and incubated for 5 min at room temperature, before 1 min centrifugation at 14,000 *g*. The elution step was repeated. Isolated RNA was subjected to DNase digestion (DNase I Set, Zymo Research, Irvine, CA, United States) and cleaned up using the RNA Clean & Concentrator kit (Zymo Research, Irvine, CA, United States). RNA concentration, integrity, and purity were assessed using 2100 Bioanalyzer and RNA 6000 Nano Kit (Agilent Technologies, Santa Clara, CA, United States). At this point, one leaf sample from the M4 salinity treated group was excluded from further analysis due to insufficient quality. Library preparation for mRNA Illumina HiSeq 4000 sequencing, as well as preprocessing to remove adapter sequences and low-quality reads were provided by Novogene (Hong Kong).

### RNA-Seq Data Analysis

The obtained 150 bp paired-end reads were trimmed to remove low-quality bases (Phred < 20), clipped to remove remaining adapter sequences, and mapped to the 12X.2 version of the PN40024 grapevine reference genome (Canaguier et al., 2017) using “CLC Genomics Workbench 12.0” (Qiagen, Hilden Germany), with the following parameters: mismatch cost 2, insertion or deletion cost 3, length fraction 1, similarity fraction 0.95, and a maximum number of hits for a read 1. The reads were annotated using the VCost.v2 annotation. Raw counts of transcripts were exported and deposited to ENA (European Nucleotide Archive) under project accession number PRJEB44658.

Normalization of the raw counts and differential expression analysis was performed in “R v3.6.3” (R Core Team, 2017), using the *limma* package v3.42.2 (Ritchie et al., 2015) with the method previously described by Dermastia et al. (2021). In short, mRNA counts with a baseline expression level of at least 50 reads mapped in at least three samples were TMM-normalized in edgeR v3.28.1 (Robinson et al., 2009) and transformed using voom (Law et al., 2014). Principal component analysis (PCA) and hierarchical clustering analysis were performed on the resulting normalized counts. PCA was performed with the pc package and hierarchical clustering analysis was performed using the “pheatmap package v 1.0.12,” applying 1-Pearson correlation as distance measure and Complete Linkage as the linkage method. Differential expression was obtained by contrasts. Gene Set Enrichment Analysis (GSEA) was performed as described by Subramanian et al. (2005) on normalized log-transformed expression data. Results with a false discovery rate FDR *q* < 0.25 were considered statistically significant.

### Targeted Gene Expression Analysis by qPCR

Differential expression of three genes, *NCED1* (*Vitvi19g01356*), *MAPK2* (*Vitvi16g01160*), *LOX* (*Vitvi06g00158*), and *UBI_CF* (*Vitvi19g00744*) as a reference gene was confirmed by qPCR. The primers and probes used are listed in Supplementary Table 1. Reverse transcription was performed with the High-Capacity RNA-to-cDNA™ kit (Applied Biosystems, Waltham, MA, United States). Power SYBR™ Green PCR Master Mix was used for all assays. The following thermal cycle conditions were applied for PCR: 95°C for 10 min, 40 cycles of 95°C for 15 s, and 60°C for 1 min; and a climb in increments of 0.05°C from 60 to 95°C for the high-resolution melting curve. The Cq values were used for relative calculation of the initial target number from a serial dilution curve using quantGenius (Baebler et al., 2017). Then, the normalized logFC values were correlated to the values obtained from the RNA-Seq analysis by Pearson correlation coefficient.

## Results

### Vine Physiology and Nutritional Status

The experimental season was warmer and drier than average. From DOY 1 to 233, the ET_o_ and rainfall were 901 and 126 mm, respectively. All rainfall events greater than 10 mm occurred before the start of irrigation (DOY 133). On DOY 233, when vine water relations and leaf gas exchange were measured and the berry and leaf samples were collected, the average air temperature was 23.6°C and the relative humidity was 70%. On that day an ET_o_ of 5 mm was recorded.

**FIGURE 1:**
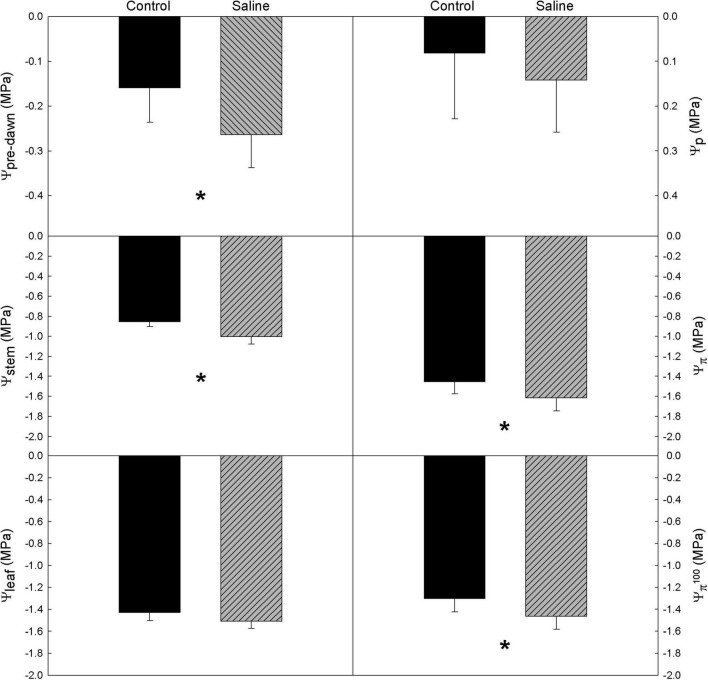
Average values of vine water relations in a Tempranillo vineyard grafted onto M1, M4, and 1103-Paulsen (1P) rootstocks subjected to different water quality (C, control and S, saline irrigation) on DOY 233 of 2019 in Valencia, Spain. Ψ_pre–dawn_, pre-dawn leaf water potential; Ψ_stem_, midday stem water potential; Ψ_leaf_, midday leaf water potential; Ψ_p_, leaf turgor potential; Ψ_π_, leaf osmotic potential; Ψ_π_^100^, leaf osmotic potential at full turgor. Data are averages and standard errors of 9 measurements per water quality. Within each parameter, an asterisk denotes significant differences between treatments at *p* < 0.05 (Duncan test).

In general, the water relations of grapevine cv. ‘Tempranillo’ was significantly affected only by water quality (WQ) (Table 1), so water potential values are plotted by water quality treatment (Figure 1). According to the Ψ_pre–dawn_ and Ψ_stem_ measurements, the WQ exerted a significant effect on the vine water status at both maximum hydration and maximum water demand with no differences among rootstocks (Figure 1). Specifically, the vines from the saline treatments exhibited more negative values than the controls. These differences were –0.12 and –0.17 MPa on average for, respectively, Ψ_pre–dawn_ and Ψ_stem_. Therefore, the effects of WQ on the water status at the time of maximum hydration (Ψ_pre–dawn_), were fairly maintained at the time of maximum evaporative demand (Ψ_stem_).

**TABLE 1:** Significance of the factor effects in the two-way ANOVAs carried out for water relations and gas exchange parameters assessed in the Tempranillo cv. vines grafted onto M1, M4, and 1103-Paulsen rootstocks.

Type of parameter	Parameter	Factors		Interaction
		Rootstock	Water Quality	R × WQ
Water relations	Ψ_pre–dawn_	0.33	**<0.001**	0.44
	Ψ_stem_	0.06	**<0.001**	0.62
	Ψ_leaf_	0.10	0.19	0.97
	Ψ_π_	0.60	**0.03**	0.46
	Ψ_p_	0.25	0.37	0.88
	Ψ_π_^100^	0.29	**0.02**	0.86
Gas exchange	A_N_	**<0.01**	**<0.001**	0.17
	*g*_s_	0.23	**0.02**	0.37
	WUE_i_	0.25	0.07	0.67

*Ψ_pre–dawn_, pre-dawn leaf water potential; Ψ_stem_, midday stem water potential; Ψ_leaf_, midday leaf water potential; Ψ_π_, leaf osmotic potential; Ψ_p_, leaf turgor potential; Ψ_π_^100^, leaf osmotic potential at full turgor; A_N_, net photosynthesis; g_s_, stomatal conductance; WUE_i_, intrinsic water use efficiency. Significance of effects in bold denotes statistically significant differences at p < 0.05.*

According to the Ψ_π_ and Ψ_π_^100^ measurements, neither the R nor the R × WQ had significant effects on the osmotic potential (Figure 1). Despite this, the vines from the saline treatments exhibited significantly more negative values than the controls. These differences were –0.16 MPa on average for both Ψ_π_ and Ψ_π_^100^. Both the Ψ_leaf_ and Ψ_p_ were unaffected by either WQ, R, or R × WQ.

**FIGURE 2:**
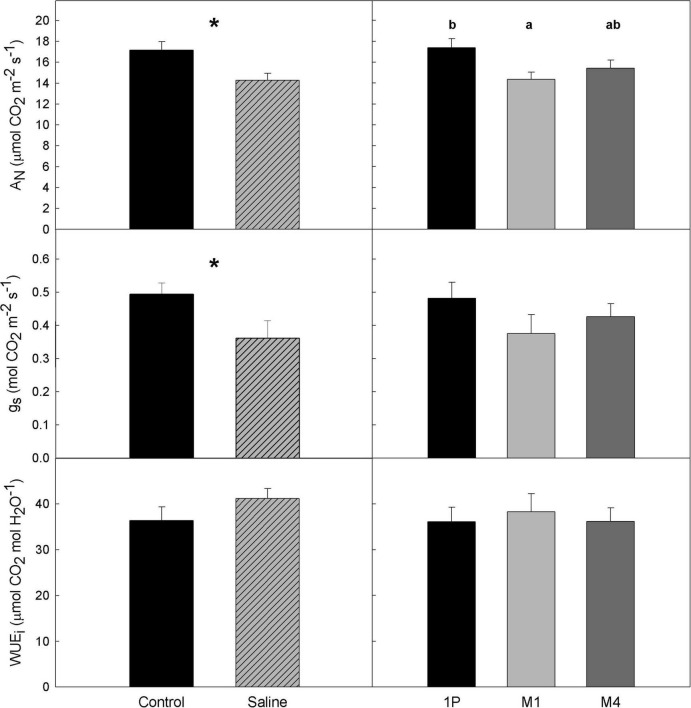
Average values of gas exchange parameters in a Tempranillo vineyard grafted onto M1, M4, and 1103-Paulsen (1P) rootstocks subjected to different water quality (C, control and S, saline irrigation) on DOY 233 of 2019 in Valencia, Spain. A_*N*_, net photosynthesis; g_*s*_, stomatal conductance; WUE_*i*_, intrinsic water use efficiency. Data are averages and standard errors of 18 and 12 measurements per water quality and rootstock, respectively. Within each parameter, asterisks or letters denote significant differences between water quality treatments or rootstocks at *p* < 0.05 (Duncan test), respectively.

Regarding gas exchange parameters, both net photosynthesis rate (A_*N*_) and leaf stomatal conductance (g_*s*_) was significantly affected by WQ, and A_*N*_ also by R (Table 1), whereas the R × WQ interactions were non-significant. Specifically, the vines from the Saline treatments presented lower values than the controls for both parameters with an average A_*N*_ value of 14.3 and 17.2 μmol CO_2_ m^–2^ s^–1^, respectively, and with average *g*_*s*_ values of 0.362 and 0.493 mol H_2_O m^–2^ s^–1^. Despite these differences in carbon assimilation and stomatal conductance rates, no significant differences in intrinsic water use efficiency (WUE_*i*_) in response to WQ were observed. Moreover, net photosynthetic rates of vines on 1103P were significantly higher than those on M1 (Figure 2).

The LAI was significantly affected by WQ (Table 2) due to reductions in the leaf area of lateral shoots (data not shown). Overall, the Saline treatments reduced the LAI per vine by 15% compared to the controls. This decreasing effect of WQ on the LAI was observed on the vines grafted onto the M-series rootstocks, mainly onto the M1. The concentrations of the macro- and micronutrients in the vine leaves were, overall, significantly affected by both WQ and R, and even by the R × WQ interaction (Table 2), which points toward an interesting rootstock salt-stress modulating effect. On the one hand, the leaf concentrations of Cl^–^, Ca^2+^, K^+^, and Mg^2+^ depended on WQ, while N and Na^+^ did not. On the other hand, the leaf concentrations of N, Cl^–^, Ca^2+^, Na^+^, and Mg^2+^ depended on R, while K^+^ did not.

**TABLE 2:** Leaf area index (LAI) and leaf nutritional status in leaf blades from *Vitis vinifera* (L.) cv. Tempranillo grafted onto M1, M4 and 1103-Paulsen (1P) rootstocks subjected to different water quality (C; control and S, saline irrigation) on DOY 233 of 2019 in Valencia, Spain.

Factors	Treatment	LAI (m^2^ m^–2^)	N (g 100g^–1^)	Cl (g 100g^–1^)	Ca (g 100g^–1^)	K (g 100g^–1^)	Na (g 100g^–1^)	Mg (g 100g^–1^)	K/Ca	K/Na
R	1P	1.8	2.26b	0.75a	2.01a	0.73	0.003a	0.41ab	0.37b	353.0b
	M1	1.8	2.12ab	1.35b	2.36b	0.65	0.004b	0.39a	0.28a	185.5a
	M4	1.9	2.07a	1.24b	1.94a	0.66	0.003a	0.46b	0.35ab	260.8ab
WQ	Control	2.0b	2.15	0.67a	1.93a	0.74b	0.003	0.40a	0.40	287.7a
	Saline	1.7a	2.15	1.54b	2.28b	0.61a	0.003	0.44b	0.44	245.1b
Interaction	1P C	1.8	2.33	0.54	1.9	0.77	0.004abc	0.40	0.42	313.7
R × WQ	1P S	1.8	2.19	0.94	2.1	0.68	0.002a	0.42	0.32	392.3
	M1 C	2.1	2.08	0.79	2.2	0.72	0.004bc	0.36	0.33	235.3
	M1 S	1.5	2.16	1.89	2.5	0.59	0.005c	0.43	0.24	135.6
	M4 C	2.0	2.04	0.68	1.7	0.74	0.003ab	0.44	0.44	314.2
	M4 S	1.8	2.10	1.79	2.2	0.58	0.003abc	0.48	0.27	207.3
Rootstock		0.89	**0.04**	**<0.01**	**<0.001**	0.25	**0.02**	**0.05**	**0.04**	**0.05**
Water Quality		**0.03**	0.99	**<0.001**	**<0.001**	**<0.01**	0.98	**0.04**	** < 0.001**	0.42
R × WQ		0.08	0.33	0.09	0.49	0.72	**0.05**	0.61	0.49	0.27

Data are averages of 6, 9, and 3 determinations per rootstock, water quality and rootstock per water quality respectively. For each parameter, letters denote significant differences between treatments at p < 0.05 (Duncan test). The statistical significance effect of the rootstock (R), water quality (WQ) and their interaction are also indicated by means of the p-values from the ANOVAs. Significance of effects in bold denotes statistically significant differences at p < 0.05.

Nitrogen was significantly higher in the vines grafted onto the 1103P than in those grafted onto the M4 (Table 2). Specifically, the Cl^–^ concentration in the leaves increased 2.3-fold on average from the controls to the saline treatments. Interestingly, this increase in leaf Cl^–^ concentration from the controls to the saline treatments was significant in the M-series rootstocks, but not in the 1103P. The Ca^2+^ concentration in the leaves also increased significantly from the controls to the saline treatments and, similarly to Cl^–^, more markedly onto the M-series than onto the 1103P (Table 2). Regarding the leaf K^+^ concentrations, the effect of WQ was also significant, leading to lower K^+^ concentrations from the controls to the saline treatments. Regarding leaf Na^+^, there were no significant differences in the concentrations in response to WQ, but there were depending on the rootstock and, interestingly enough, depending on the R × WQ interaction. Specifically, the M1 tended to accumulate Na^+^ in the leaves in response to the Saline treatments, which is an effect not observed for 1103P or M4 (Table 2). Thus, the M1 showed the lowest K^+^/Ca^2+^ ratio and the K^+^/Na^+^ one. Finally, there were differences in leaf Mg^2+^ concentrations in response to both WQ and R, which were statistically, but, maybe, not practically significant (Table 2).

### Grape Composition

The grape composition was less affected by WQ than by Ress, some statistically significant interactions between both factors were observed (Table 3). The TSS was affected by WQ and R and, in addition, the effect of WQ significantly changed in magnitude from one rootstock to the others, i.e., the interaction R × WQ was also significant. Specifically, grape TSS tended to increase from the controls to the saline treatments with a greater increment in the vines onto the M1 rootstock (Table 3). Contrary to TSS, the other grape technological composition parameters (pH, TA) were neither affected by R nor WQ nor R × WQ (Table 3).

**TABLE 3:** Parameters of grape composition at harvest for Tempranillo wine grapes grafted onto M1, M4, and 1103-Paulsen (1P) rootstocks subjected to different water quality (C; control and S; saline irrigation) in Valencia, Spain.

Factors	Treatment	Berry weight (g)	TSS (°)	T.A. (g L^–1^)	pH	Anthocyanins (mg g^–1^)	Polyphenols (mg g^–1^)
R	1P	1.6	20.1a	3.8	4.17	0.74c	4.83b
	M1	1.6	20.6b	3.3	4.17	0.53b	4.08a
	M4	1.7	20.9b	3.6	4.16	0.44a	3.73a
WQ	Control	1.7	20.2a	3.5	4.17	0.60	4.34
	Saline	1.6	20.8b	3.7	4.16	0.54	4.08
Interaction R × WQ	1P C	1.67	19.9a	3.7	4.15	0.83	5.14d
	1P S	1.61	20.2ab	3.9	4.19	0.65	4.51c
	M1 C	1.64	19.8a	3.2	4.22	0.55	4.35bc
	M1 S	1.51	21.4c	3.4	4.12	0.52	3.81a
	M4 C	1.70	20.9bc	3.5	4.15	0.42	3.54a
	M4 S	1.60	20.8bc	3.7	4.16	0.45	3.92ab
Rootstock		0.75	**<0.01**	0.19	0.96	**<0.001**	**<0.001**
Water Quality		0.29	**<0.01**	0.38	0.70	0.13	0.07
R × WQ		0.94	**<0.01**	0.99	0.30	0.09	**0.02**

*TSS, total soluble solids; T.A., titratable acidity. Data are averages of 6, 9, and 3 determinations per rootstock, water quality and rootstock per water quality respectively. Within each parameter, letters denote significant differences between treatments at p < 0.05 (Duncan test). The statistical significance effect of the rootstock (R), water quality (WQ) and their interaction are also indicated by means of the p-values from the ANOVAs. Significance of effects in bold denotes statistically significant differences at p < 0.05.*

Regarding the phenolic composition, i.e., anthocyanins and polyphenols contents, it was not significantly affected by WQ, but heavily depended on R. Besides, a significant R × WQ interaction was also revealed in the polyphenols, which points toward an interesting change in the effect of WQ depending on the rootstock (Table 3). Specifically, both the polyphenols and the anthocyanins contents tended to decrease from the controls to the saline treatments onto the 1103P and on M1, with no changes onto the M4 (Table 3). Regardless of the effect of WQ on phenolic composition in grapes, the 1103P tended to have higher anthocyanins and polyphenols than the other two rootstocks.

### Differential Gene Expression

High-throughput mRNA sequencing was performed on whole leaf and berry skin samples from cv. ‘Tempranillo’ was grafted onto the three different rootstocks and exposed to salinity stress. On average, 41,326,458 reads were mapped in pairs to the grapevine genome. Of the 42,413 genes annotated in grapevine, 16,790 were expressed in sufficient quantities for statistical analysis.

**FIGURE 3:**
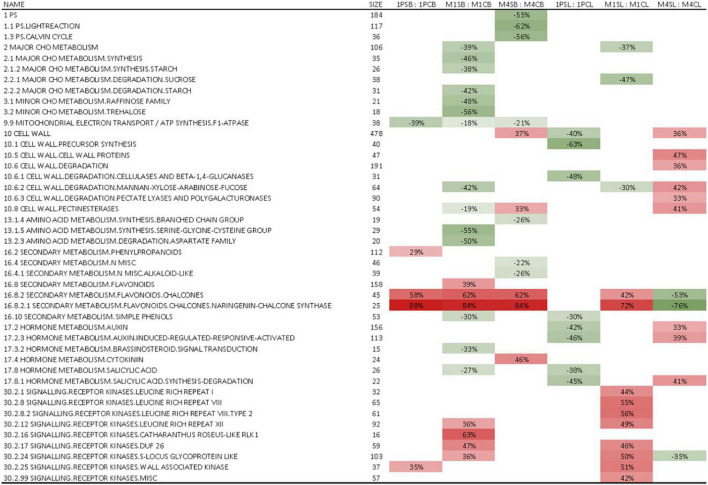
A subset of significantly enriched gene sets obtained by GSEA (The full set is presented in Supplementary Table 2). Values represent the percentages of genes that were positively (+) or negatively (–) regulated within a particular bin in leaves and berries of cv. ‘Tempranillo’ vines grafted onto 1103-Paulsen, M1, or M4 rootstocks subjected to salinity irrigation. Only statistically significant (FDR *q* value < 0.25) values are shown. Red denotes positive enrichment or upregulation and green denotes negative enrichment or downregulation. C, control; S, salinity; 1P, 1103-Paulsen rootstock; M1, M1 rootstock; M4, M4 rootstock; L, leaves; B, berries.

**FIGURE 4:**
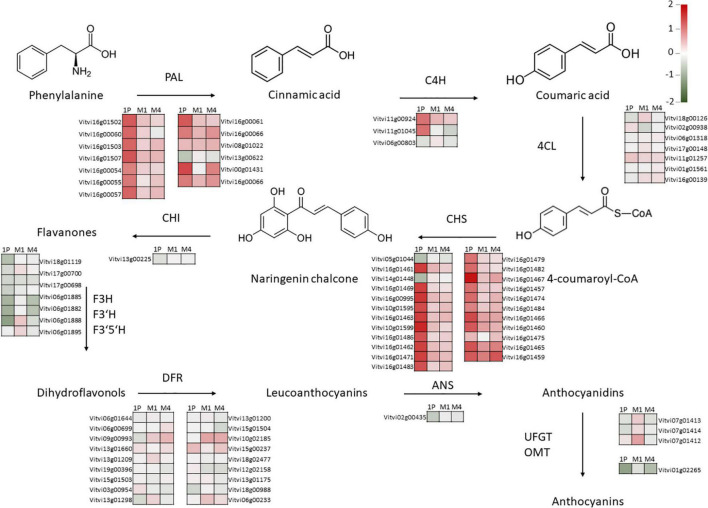
Log_2_ FC values of genes involved in anthocyanin synthesis in Tempranillo in salinity-treated berries as compared to controls grafted onto 1103-Paulsen, M1, and M4. The specific gene names are provided by means of the Vitvi identifiers. Color represents the value of Log_2_ FC. PAL – phenylalanine ammonia-lyase; C4H, cinnamate-4-hydroxylase; C4L, 4-coumarate: CoA ligase; CHS, chalcone synthase; CHI, chalcone-flavanone isomerase; F3H, flavanone 3-hydroxylase; F3′H, flavonoid 3′-hydroxylase; F3′5′H, flavonoid 3′5′-hydroxylase; DFR, dihydroflavonol 4-reductase; ANS, anthocyanin synthase; UFGT, anthocyanidin 3-*O*-glucosyltransferase; OMT, *O*-methyltransferase; 1P, 1103-Paulsen rootstock; M1, M1 rootstock; M4, M4 rootstock.

Although hierarchical clustering analysis and PCA of leaf and berry skin samples showed no apparent correlation in gene expression regarding either the WQ or R and no clear clustering was observed on PCA for either tissue (Supplementary Figures 1, 2), GSEA identified several processes (bins) that were statistically significantly (FDR *q* < 0.25) differentially expressed due to WQ in leaves and berries of scions grafted on the three rootstocks (Figure 3). The number of significantly differentially expressed bins was higher in leaves and berries of scions grafted on M4 and M1 rootstocks as compared to 1103P. The strongest enrichment was detected for flavonoid synthesis bins in berry skins for all three R. In them, chalcone synthases contribution prevailed (Supplementary Table 2). When examining the expression of individual genes involved in this pathway, large differences in average values were observed, with up to a fourfold difference in a uniform dominant upregulation pattern, although no statistically significant differences in gene expression were found between the control and saline treatments (Supplementary Table 3). Specifically, the differences in average values between salt-stressed and control vines were the highest in the expression of genes related to chalcone synthase (CHS) and phenylalanine ammonia-lyase (PAL) genes. This was most apparent in berry skins, where most of the PAL and CHS genes showed an upregulation pattern due to WQ (Figure 4). Moreover, the differences were highest in vines grafted onto 1103P than onto M4 and M1. However, multiple flavanone 3-hydroxylases showed a downregulation pattern in these samples. On the other hand, leaf samples showed lower differences, which were found in CHS genes in samples grafted onto M1, and some flavanone 3-hydroxylase genes in samples grafted onto M4 (Supplementary Figure 3).

Although no statistically significant differences in expression of individual genes were observed due to WQ in either leaves or the berries, some statistically significant differences due to R were observed (Supplementary Table 3). There were 15 differentially expressed genes found between the leaves of control plants grafted onto 1103P and M4. Most of them were more expressed in 1103P than in M4, but no specific pathway predominated among them.

The technical validity of RNA-Seq and the data analysis pipeline was corroborated by the targeted analysis of three genes by qPCR. The qPCR results highly correlated with RNA-Seq (*r*^2^ = 0.83) (Supplementary Figure 4).

## Discussion

The effects of WQ and R on physiology and transcriptomics of cv. ‘Tempranillo’ vines were assessed indirectly because all determinations were carried out on the scion, not in the rootstock, which is the barrier against soil salinity. However, the scion cultivar is the genotype that ultimately bears fruit and ripens it and, therefore, confers economic value on the crop (Marguerit et al., 2012). Thus, in this approach, the scion is considered an integrator of the effects induced by the rootstock. It is important to bear this in mind when interpreting the results, especially the transcriptome analyses, because of the combination of two *Vitis* spp. Genotypes are studied by evaluating only one of them, i.e., *Vitis vinifera* L. In comparison, most of the grapevine transcriptomics responses reported in the literature have been assessed on a single genotype, i.e., directly in the own-rooted *Vitis vinifera* (Cramer et al., 2007; Guan et al., 2018; Das and Majumder, 2019; Lehr et al., 2022) or on the rootstock without grafting (Gong et al., 2011; Henderson et al., 2014; Meggio et al., 2014; Corso et al., 2015; Vannozzi et al., 2017; Fu et al., 2019; Çakır Aydemir et al., 2020), and if carried out in both the scion and the rootstock, they have been under highly controlled conditions (Upadhyay et al., 2018; Bianchi et al., 2020; Franck et al., 2020; Baggett et al., 2021), i.e., not under real field-grown conditions.

In the present trial, the water requirements of the grapevines were fully met trying to isolate the effect of WQ on the physiological and transcriptomic responses. When plant measurements and samplings were carried out, the water status experienced by the control vines grafted onto any of the rootstocks was indicative of very mild water stress according to Williams and Baeza (2007; Figure 1). This implies that irrigation largely met the evapotranspiration demand of the plants. However, it was not excessive, which would have resulted in irrigation water percolation and thus the washout of salts from the rooting depth. In fact, the ions’ concentration in the soil solution of Saline treatments caused vine water stress. This was observed in the general decrease of both Ψ_pre–dawn_ and Ψ_stem_ in the vines grafted onto all rootstocks under irrigation with saline water, which means a worsening of the plant water status (Figure 1). This physiological response is likely due to a reduction of the soil water potential by an osmotic effect (Tattersall et al., 2007), i.e., the so-called osmotic drought (Chaves et al., 2009). As expected, Ψ_pre–dawn_ was in line with Ψ_stem_ (Suter et al., 2019), although plants onto M4 tended to show less negative Ψ_stem_ values than those onto 1103P, with no difference in Ψ_pre–dawn_ (Table 1). These slight differences in Ψ_stem_ between M4 and 1103P agreed with what Frioni et al. (2020) observed in M4 under water shortage.

Plants react to salt stress and control their subsequent physiological responses using signals, which can be ionic, osmotic, hormonal, and/or reactive oxygen species regulation (Shahid et al., 2020; Zhou-Tsang et al., 2021). Concerning the ionic, in this work the leaf ion concentrations have been observed to differ among rootstocks, notably, Cl^–^, Ca^2+^, Na^+^, and Mg^2+^ (Table 2). Regarding Cl^–^, it usually builds up in the leaves of woody crops, and the plant’s ability to avoid accumulating Cl^–^ in leaves is considered directly proportional to its salinity tolerance. In this work the M-series rootstocks increased the leaf Cl^–^ twofold in the saline treatment compared to the control. In contrast, in the 1103P the leaf Cl^–^ increase in the saline treatment compared to the control was not significant. These results are in agreement, on the one hand, with Meggio et al. (2014), who also reported higher leaf Cl^–^ in vines onto M4 in comparison to the good salt excluder 101–14 Mgt (Walker et al., 2004, 2010) and, on the other hand, with Urdanoz and Aragüés (2009), who reported that the ‘Tempranillo’ cultivar grafted onto 1103P was able to exclude Cl^–^ from the leaves more efficiently than other cultivar-rootstock combinations.

The leaf Cl^–^ non-accumulation ability conferred by the 1103P could be due to (i) limited salt uptake, i.e., ion exclusion, and (ii) limited salt translocation from the root to the shoot. Abbaspour et al. (2013) suggested that 1103P contributes to reducing shoot Cl^–^ concentration by root efflux and vacuolar internalization. Besides, Henderson et al. (2014) suggested that transcriptional events contributing to the Cl^–^ exclusion mechanism in grapevine are not stress-inducible, but constitutively different between contrasting genotypes. Anyway, Cl^–^ exclusion factors are yet to be identified at the transcriptomic level, and are multigenic, including transport proteins (Gong et al., 2011; Das and Majumder, 2019; Zhou-Tsang et al., 2021). This genotype-dependent, though fuzzy, transcriptomic effects agree with our GSEA results, which identified much less statistically significantly (FDR *q* < 0.25) differentially expressed bins due to WQ in ‘Tempranillo’ grafted onto 1103P as compared to M4 and M1 (Figure 3). Baggett et al. (2021), also similarly observed that salinity affected transcript abundance more in salt-sensitive genotypes than in salt-tolerant ones. Importantly, the leaf Cl^–^ concentrations in our trial are higher than the ones reported by Urdanoz and Aragüés (2009) and Baggett et al. (2021), even though in the range of the ones found in ‘Cabernet Sauvignon’ onto 1103P by Dag et al. (2015) using similar WQ.

The capacity of rootstocks to restrict leaf salt buildup should not be the only parameter for rootstock selection (Zhou-Tsang et al., 2021). Regarding other criteria, several authors indicated the better M4 performance compared to other rootstocks because of an improved antioxidant capability (Meggio et al., 2014; Corso et al., 2015; Lucini et al., 2020; Prinsi et al., 2020). Furthermore, it is important to consider the likely accumulation of Cl^–^ and Na^+^ in the permanent instead of the short-lived organs of the vine (Stevens and Partington, 2013; Netzer et al., 2014), which may lead to salinity carry-over effects on the medium-to-long term. Based on our results, this would be a concern for rootstocks M1 and M4 and less for 1103P (Table 1), because of its possible detrimental effects on future bud fruitfulness (Walker et al., 2002). In fact, Dag et al. (2015) reported that irrigating the ‘Cabernet-Sauvignon’ scion grafted onto 1103P with water similar in salinity to the Saline treatment in this work, did not significantly affect vine performance in the first two seasons, but that Na^+^ and Cl^–^ accumulation in the wood eventually led to vine death in the third one.

Regarding Na^+^, it is less prone to build up in the leaves of grapevines than Cl^–^ (Henderson et al., 2018), which, given the Na^+^/Cl^–^ ratio of the waters applied in this work, was also observed here (Table 2). However, there were differences in salt-stress modulating ability among rootstocks with the M1 more liable to leaf Na^+^ accumulation as salinity increased than 1103P or M4. Regarding leaf Ca^2+^, it increased in the Saline treatments compared to the Controls (Table 2). That leaf Ca^2+^ increased in the ‘Tempranillo’ leaves as salinity grew regardless of the rootstock suggests that all three rootstocks can maintain high Ca^2+^/Na^+^ ratios and thus, efficiently exclude Na^+^ (Shahid et al., 2020). More interestingly, however, there were differences in leaf Ca^2+^ among the vines depending on the rootstock. Particularly, the M1 built up significantly more leaf Ca^2+^ than the 1103P and M4 (Table 2). Since Ca^2+^ can regulate plant signaling, enzyme activity, ion channel performance, and gene expression (Golldack et al., 2014), the higher leaf Ca^2+^ onto the M1 may be a positive plant adaptation as previously reported by Porro et al. (2013). Likewise, K^+^ is also key in maintaining the osmotic balance and thus the ionic homeostasis in plant cells (Kumari et al., 2015; Guan et al., 2018). However, in our work, leaf K^+^ decreased because of salinity, without differences among rootstocks (Table 2). Similarly, Guan et al. (2018) also found a decreasing trend in leaf K^+^ in ‘Summer Black’ cv. in response to NaCl irrigation, and Munns and Tester (2008) indicated that a strong relationship between leaf K^+^ and salt tolerance had not yet been reported. In our work, both the leaf K^+^/Ca^2+^ and K^+^/Na^+^ ratios were reduced by M1 compared to 1103P. This suggests that the 1103P conferred a greater salinity tolerance to the scion than the M1.

Concerning the osmolyte regulation signals, a tendency to a slight osmotic adjustment was observed in the leaves on all three rootstocks. This is because, independently of the leaf water status, i.e., Ψ_π_^100^, the values of the saline treatments were significantly more negative (–0.16 MPa on average) than those of the Controls (Figure 1). Through osmotic adjustment plants cope with declining soil water potential mainly because increasing osmolyte concentrations decrease the water potential within plant cells, thus increasing the leaf relative water content and turgor for a given soil water potential (Barrios-Masias et al., 2018). These osmolytes can be inorganic, which are actively and passively taken from the same soil solution, or organic, which are obtained by biosynthesis of proline, glycine-betaine, etc. However, in our work, the expression of genes involved in amino acid metabolism was not altered in leaves in response to WQ (Figure 3), whereas the concentration of Cl^–^, K^+^, and Ca^2+^ did increase in the leaves (Table 2). Accordingly, the slight observed osmotic adjustment was achieved through the build-up of inorganic osmolytes, and this was controlled by the rootstock because the root is the organ that regulates the entry of the soil solution ions into the plant. The mechanisms of ion exclusion and/or upward movement along the xylem should be genetically regulated at the root level, i.e., over-expression of the cation HKT transporters genes (Deinlein et al., 2014; Fu et al., 2019; Zhou-Tsang et al., 2021), and not at the scion level. However, despite occurring at the root level, the mechanisms may be genetically regulated in a scion-induced manner (Franck et al., 2020) and then, maybe, detected in the scion. Remarkably, among the 15 differentially expressed genes between the 1103P and M4, a lactoylglutathione lyase (Vitvi04g01424) and a Dof family transcription factor (Vitvi18g00858) were found. These genes have previously been implicated in response to abiotic stress in grapevine (Shangguan et al., 2020), as it was implicated in redox homeostasis in heat-stressed ‘Muscat Hamburg’ berries (Carbonell-Bejerano et al., 2013).

The generalized reduction found in net photosynthesis (A_*N*_) under saline conditions, regardless of the rootstock (Figure 2), is related to stomatal and mesophyll conductance limitation, as there were no major differences in WUE_*i*_ beyond those expected, given the differences in water status (Flexas et al., 2004). Reductions are in line with those found by Flexas et al. (1999) in ‘Tempranillo’ and Baeza et al. (2007) and Baggett et al. (2021) in ‘Cabernet-Sauvignon’ cultivars. Moreover, no differences were detected in the ratio of internal to atmospheric CO_2_ concentration (Ci/Ca) between treatments (0.76 and 0.75 in Control and Saline treatments, respectively; data not shown). This suggests that in this work salinity was not high enough to induce either toxic effects on the photosynthetic apparatus or cellular damage in the leaves, as confirmed using the leaf transcriptomic analysis (Figure 3), but rather that it simply increased water stress by lowering the soil water potential, which eventually showed up in g_*s*_ and, thus, A_*N*_ reduction (Figure 2). Interestingly, according to Bianchi et al. (2020), water shortcoming stress decreases stomatal conductance due to lower water potential, but the photosynthetic activity keeps high with bare differences among 1103P, M1, and M4. In contrast, in our trial, M1 performed differently from the other rootstocks by inducing an overall reduction in A_*N*_. Moreover, Bianchi et al. (2020) did detect changes in the transcript abundances of key genes related to abscisic acid biosynthesis, but in the root, not in leaves, and studying only the wider *Vitis* spp. genotype.

The overall effects caused by salinity on decreasing leaf photosynthesis as well as LAI (Figure 2 and Table 2) should have led to reduced berry ripening (Cramer et al., 2007; Chaves et al., 2009; Liu et al., 2020; Zhou-Tsang et al., 2021). However, the opposite was observed. The Saline treatments increased TSS compared to the Control grapes. These results point toward the ability of all rootstocks to keep allocating energy resources to fruit ripening regardless of salt stress. Interestingly, Meggio et al. (2014) also highlighted the salt tolerance of these rootstocks regardless of their ability to limit specific ion accumulations in the scion, which was associated with a lower decrease in A_*N*_ and Ψ_leaf_ on M4 compared to 101–14 Mgt. This was not observed under salinity in this work, as it neither was an underwater shortage (Bianchi et al., 2020).

Effects of WQ and R on grape composition are usually not very conclusive according to studies where both factors are combined (Walker et al., 2007; Stevens et al., 2011; Hirzel et al., 2017; Mirás-Avalos and Intrigliolo, 2017). This is because there is a multitude of environmental factors that interact with rootstock response, most notably soil type (Ferlito et al., 2020). Specifically, the three rootstocks studied here perform well on soils high in calcium carbonate like the one used in this investigation because all three come from crossings with *Vitis berlandieri*, a species that evolved on calcareous soils (Harry, 1996). In this work, there was a salt-stress modulating effect by the rootstock on grape composition, primarily TSS and, secondarily, the phenolic composition as revealed by the R × WQ interactions (Table 3). Whereas barely anything was observed on T.A., and, specifically, pH, which did not change following the decrease in leaf K^+^ concentration due to salinity (Table 2) in accordance with Marín et al. (2021). Contrary to T.A., and pH, the TSS increased onto the M1 rootstock as salinity grew, whereas the other rootstocks did not respond in the same way. Moreover, the phenolic substances were also subjected to rootstock-specific modulating effects (Table 3). Despite these, the expected changes on gene expression of CHS and PAL pathways were not observed (Figure 4). This is, the significant reduction in anthocyanins content found in 1103P vines and polyphenols found in 1103P and M1 vines in response to salinity (Table 3) could not be related to the transcriptomic changes observed, nor to differences in berry size (Table 3).

Several studies have linked ultraviolet light to the induction of phenolic compound synthesis, specifically the expression of the CHS gene, a key enzyme in flavonoid biosynthesis (Merkle et al., 1994; Hernández et al., 2009; Wang et al., 2016; Reshef et al., 2018). However, these putative changes, which are related to berry exposure to sunlight in response to the saline effect on the vine leaf area (Zarrouk et al., 2016; Torres et al., 2020), would have been offset by the slight increase in the leaf area-to-production ratio (Walker et al., 2000; Bobeica et al., 2015). Moreover, flavonoid synthase is also involved in drought and osmotic stress tolerance and is controlled by rootstocks (Dal Santo et al., 2018; Bianchi et al., 2020; Zombardo et al., 2020). For instance, Zombardo et al. (2020), also in grape skin during ripening, reported some differentially expressed genes mainly involved in the synthesis and transport of phenylpropanoids (e.g., flavonoids) in response to rootstock effects. Besides, the most prominent differences in gene expression of the anthocyanin pathway usually occur during veraison, together with the differences of anthocyanin content and profile in the berry and begin to faint as the berry reaches final maturity (Castellarin et al., 2006; Castellarin and Di Gaspero, 2007). All of this highlights the complexity of relating phenotypic observations to changes in gene expression (Fu et al., 2019; Haider et al., 2019). In this regard, the next generation of omics is expected to help to identify gene function, speeding up the rootstock breeding programs for enhancing resilience to climate change in future viticulture (Marín et al., 2021).

## Conclusion

The results of this work have shown how the grapevine M-rootstock’s physiological and transcriptomic responses integrate at the scion level because of the irrigation with saline water under real field-grown conditions for the first time. The determinations carried out in the scion (i.e., cv. ‘Tempranillo’) permitted us to obtain some insight into the possible mechanisms developed by the rootstocks in response to water salinity, and the differences between the three that were tested in this work. In the short period of this trial, and a vineyard under establishment, all three rootstocks similarly adjusted osmotic potential to cope with osmotic stress, and then, vine water status declined in response to irrigation with saline water compared to non-saline water. Regarding the differential response among rootstocks, based on, on the one hand, grapevine physiology and grape must composition and, on the other hand, salt accumulation in leaves and transcriptomic changes, there were differences worth highlighting. First, the M1 rootstock was the one that responded the most to salinity by reducing A_*N*_ and LAI, whereas the M4 rootstock was the one that buffered the best the effects of salinity on TSS and grape phenolic composition. Second, the 1103P rootstock was the one able to reduce the leaf Cl^–^ and Na^+^ build-up the most and affected transcriptomic expression the least, which might have positive effects on the long-term vine performance and grape composition. Longer-term studies are needed to unravel the molecular responses occurring in mature vineyards at both the scion and rootstock levels.

## Data Availability Statement

The datasets presented in this study can be found in online repositories. The names of the repository/repositories and accession number(s) can be found below: https://www.ebi.ac.uk/ena, PRJEB44658.

## Author Contributions

IB, JP-P, FV, DI, LB, MP-N, and JP contributed to the conception and design of the study. IB, JP-P, and RS acquired the data. IB, JP-P, FV, DI, KG, and MP-N performed the data analysis and interpretation. IB and RS prepared the first-draft. IB, JP-P, FV, RS, DI, MP-N, and JP reviewed and edited the manuscript. DI, LB, and JP supervised the work. DI, LB, MP-N, and JP acquired the funding. All authors read and approved the submitted version.

## Conflict of Interest

The authors declare that the research was conducted in the absence of any commercial or financial relationships that could be construed as a potential conflict of interest.

## Publisher’s Note

All claims expressed in this article are solely those of the authors and do not necessarily represent those of their affiliated organizations, or those of the publisher, the editors and the reviewers. Any product that may be evaluated in this article, or claim that may be made by its manufacturer, is not guaranteed or endorsed by the publisher.

## Supplementary Material

Supplementary Figure 1 Gene expression overview in berry skin samples. **(A)** Hierarchical clustering analysis of the 500 most variable genes in berry skin samples. **(B)** Principal component analysis of the berry skin samples. PC, principal component; 1P, 1103-Paulsen; C, control; S, salinity.

Supplementary Figure 2 Gene expression overview in leaf samples. **(A)** Hierarchical clustering analysis of the 500 most variable genes in leaf samples. **(B)** Principal component analysis of the berry skin samples. PC, principal component; 1P, 1103-Paulsen; C, control; S, salinity.

Supplementary Figure 3 Log_2_ FC values of genes involved in anthocyanin synthesis in Tempranillo in salinity-treatment leaves as compared to controls grafted onto 1103-Paulsen, M1, and M4. The specific gene names are provided by means of the Vitvi identifiers. Color represents the value of Log_2_ FC. PAL, phenylalanine ammonia-lyase; C4H, cinnamate-4-hydroxylase; C4L, 4-coumarate: CoA ligase; CHS, chalcone synthase; CHI, chalcone-flavanone isomerase; F3H, flavanone 3-hydroxylase; F3′H, flavonoid 3′-hydroxylase; F3′5′H, flavonoid 3′5′-hydroxylase; DFR, dihydroflavonol 4-reductase; ANS, anthocyanin synthase; UFGT, anthocyanidin 3-*O*-glucosyltransferase; OMT, *O*-methyltransferase; 1P, 1103-Paulsen rootstock; M1, M1 rootstock; M4, M4 rootstock.

Supplementary Figure 4 Correlation between RNA-Seq and qPCR differential expression represented as log_2_ FC. *R*^2^ = 0.83.

Supplementary Table 1 Primers and probes used for grapevine gene expression analysis.

Supplementary Table 2 Gene sets significantly enriched by GSEA. Values represent the percentages of genes that were positively (+) or negatively (–) regulated within a particular bin in leaves and berries of cv. Tempranillo vines grafted onto 1103-Paulsen, M1, or M4 rootstocks and subjected to salinity stress. The values are derived from comparison of control and salinity-treated plants in selected time-points. Only the values with statistical significance (FDR *q* value < 0.25) are listed. Red denotes positive enrichment or upregulation and green denotes negative enrichment or downregulation. The percent value represents the proportion of genes in the gene set which contributed to the enrichment. C, control; S, salinity; 1P, 1103-Paulsen rootstock; M1, M1 rootstock; M4, M4 rootstock; L, leaves; B, berries.

Supplementary Table 3 High-throughput RNA-Seq of salinity- treated grapevine berry skin and leaf samples cv. Tempranillo grafted onto 1103-Paulsen, M1, and M4. C, control; S, salinity; 1P, 1103-Paulsen rootstock; M1, M1 rootstock; M4, M4 rootstock; L, leaves; B, berries.
