# Corrigendum: Physiological and transcriptional responses to saline irrigation of young ‘Tempranillo’ vines grafted onto different rootstocks

**DOI:** 10.3389/fpls.2022.1011533

**Published:** 2022-09-26

**Authors:** Ignacio Buesa, Juan G. Pérez-Pérez, Fernando Visconti, Rebeka Strah, Diego S. Intrigliolo, Luis Bonet, Kristina Gruden, Maruša Pompe-Novak, Jose M. de Paz

**Affiliations:** ^1^ Instituto Valenciano de Investigaciones Agrarias, Centro para el Desarrollo de la Agricultura Sostenible, Unidad Asociada al CSIC “Riego en la AgriculturaMediterránea”, Valencia, Spain; ^2^ Ecophysiologie et Génomique Fonctionnelle de la Vigne, Institut National de la Recherche Agronomique, Institut des Sciences de la Vigne et du Vin, Villenave d’Ornon, France; ^3^ Research Group on Plant Biology Under Mediterranean Conditions, Department of Biology, University of the Balearic Islands, Palma, Spain; ^4^ Centro de Investigaciones sobre Desertificación, Departmento de Ecología (CSIC, UV, GV), Valencia, Spain; ^5^ Department of Biotechnology and Systems Biology, National Institute of Biology, Ljubljana, Slovenia; ^6^ Jožef Stefan International Postgraduate School Ljubljana, Ljubljana, Slovenia; ^7^ School for Viticulture and Enology, University of Nova Gorica, Vipava, Slovenia

**Keywords:** osmotic adjustment, gas exchange, gene expression, water relations, *Vitis vinifera L.* (grapevine), salinity tolerance

In the original article, there was an error. In **Table 2** the units shown for chlorides (Cl) are not correct. To solve this problem, we include the Table transforming the units to those of the rest of the elements.

**Table 2:** Leaf area index (LAI) and leaf nutritional status in leaf blades from *Vitis vinifera* (L.) cv. Tempranillo grafted onto M1, M4 and 1103-Paulsen (1P) rootstocks subjected to different water quality (C; control and S; saline irrigation) on DOY 233 of 2019 in Valencia, Spain.

Factors	Treatment	LAI (m^2^ m^-2^)	N (g 100g^-1^)	Cl (g 100g^-1^)	Ca (g 100g^-1^)	K (g 100g^-1^)	Na (g 100g^-1^)	Mg (g 100g^-1^)	K/Ca	K/Na
R	1P	1.8	2.26b	0.75a	2.01a	0.73	0.003a	0.41ab	0.37b	353.0b
	M1	1.8	2.12ab	1.35b	2.36b	0.65	0.004b	0.39a	0.28a	185.5a
	M4	1.9	2.07a	1.24b	1.94a	0.66	0.003a	0.46b	0.35ab	260.8ab
WQ	Control	2.0b	2.15	0.67a	1.93a	0.74b	0.003	0.40a	0.40	287.7a
	Saline	1.7a	2.15	1.54b	2.28b	0.61a	0.003	0.44b	0.44	245.1b
Interaction R × WQ	1P C	1.8	2.33	0.54	1.9	0.77	0.004abc	0.40	0.42	313.7
	1P S	1.8	2.19	0.94	2.1	0.68	0.002a	0.42	0.32	392.3
	M1 C	2.1	2.08	0.79	2.2	0.72	0.004bc	0.36	0.33	235.3
	M1 S	1.5	2.16	1.89	2.5	0.59	0.005c	0.43	0.24	135.6
	M4 C	2.0	2.04	0.68	1.7	0.74	0.003ab	0.44	0.44	314.2
	M4 S	1.8	2.10	1.79	2.2	0.58	0.003abc	0.48	0.27	207.3
Rootstock		0.89	**0.04**	**<0.01**	**<0.001**	0.25	**0.02**	**0.05**	**0.04**	**0.05**
Water Quality		**0.03**	0.99	**<0.001**	**<0.001**	**<0.01**	0.98	**0.04**	**<0.001**	0.42
R × WQ		0.08	0.33	0.09	0.49	0.72	**0.05**	0.61	0.49	0.27

Data are averages of 6, 9 and 3 determinations per rootstock, water quality and rootstock per water quality respectively. For each parameter, letters denote significant differences between treatments at p < 0.05 (Duncan test). The statistical significance effect of the rootstock (R), water quality (WQ) and their interaction are also indicated by means of the p-values from the ANOVAs. Significance of effects in bold denotes statistically significant differences at p < 0.05.

A correction has been made to **Table 2**:

The authors apologize for this error and state that this does not change the scientific conclusions of the article in any way. The original article has been updated.

## Publisher’s note

All claims expressed in this article are solely those of the authors and do not necessarily represent those of their affiliated organizations, or those of the publisher, the editors and the reviewers. Any product that may be evaluated in this article, or claim that may be made by its manufacturer, is not guaranteed or endorsed by the publisher.

