# Supplementary figures and images for: Physiological and Transcriptional Responses to Saline Irrigation of Young ‘Tempranillo’ Vines Grafted Onto Different Rootstocks
Source: Front Plant Sci. 2022 Jun 6;13:866053. doi: 10.3389/fpls.2022.866053 (PMC9207310; doi:10.3389/fpls.2022.866053)

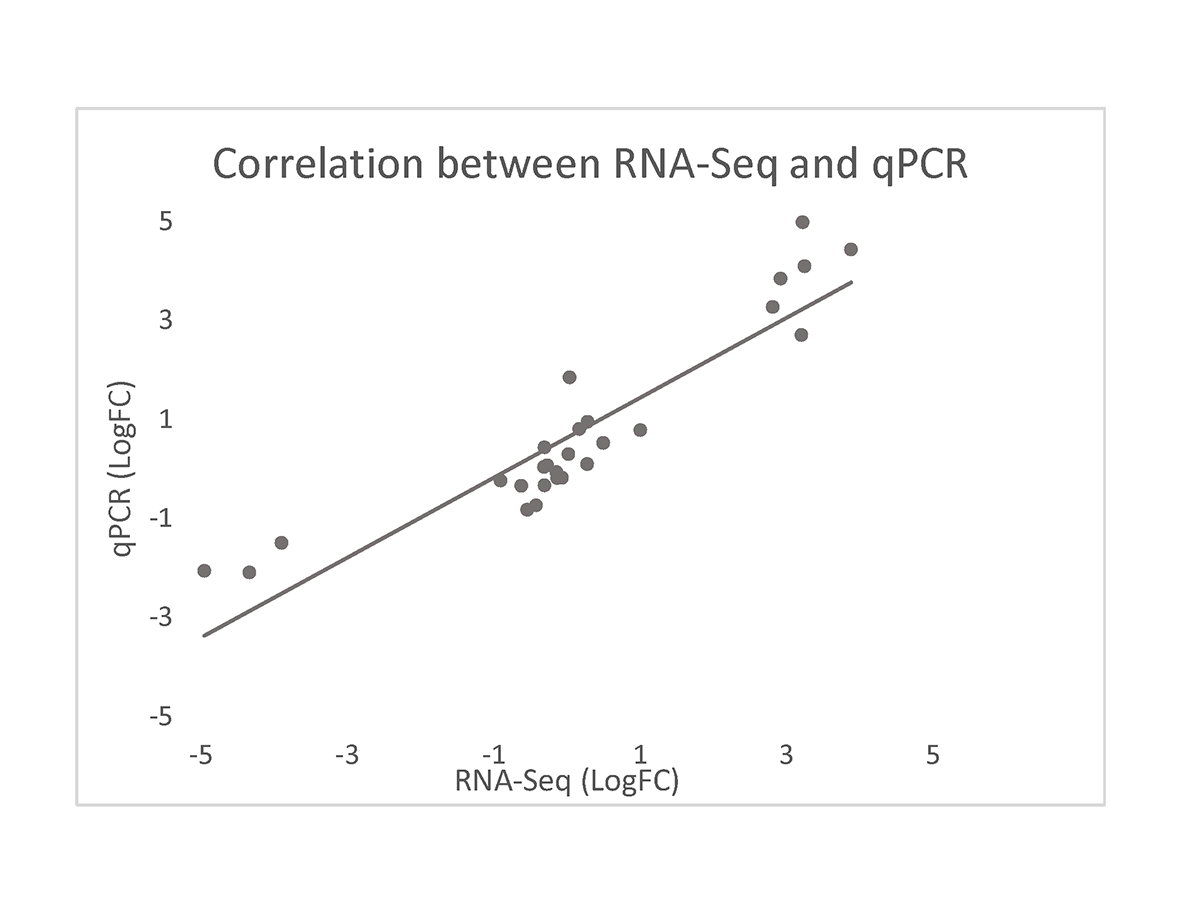

Supplement: Supplementary Figure 1 — Gene expression overview in berry skin samples. (A) Hierarchical clustering analysis of the 500 most variable genes in berry skin samples. (B) Principal component analysis of the berry skin samples. PC, principal component; 1P, 1103-Paulsen; C, control; S, salinity. [file Image_1.tif]

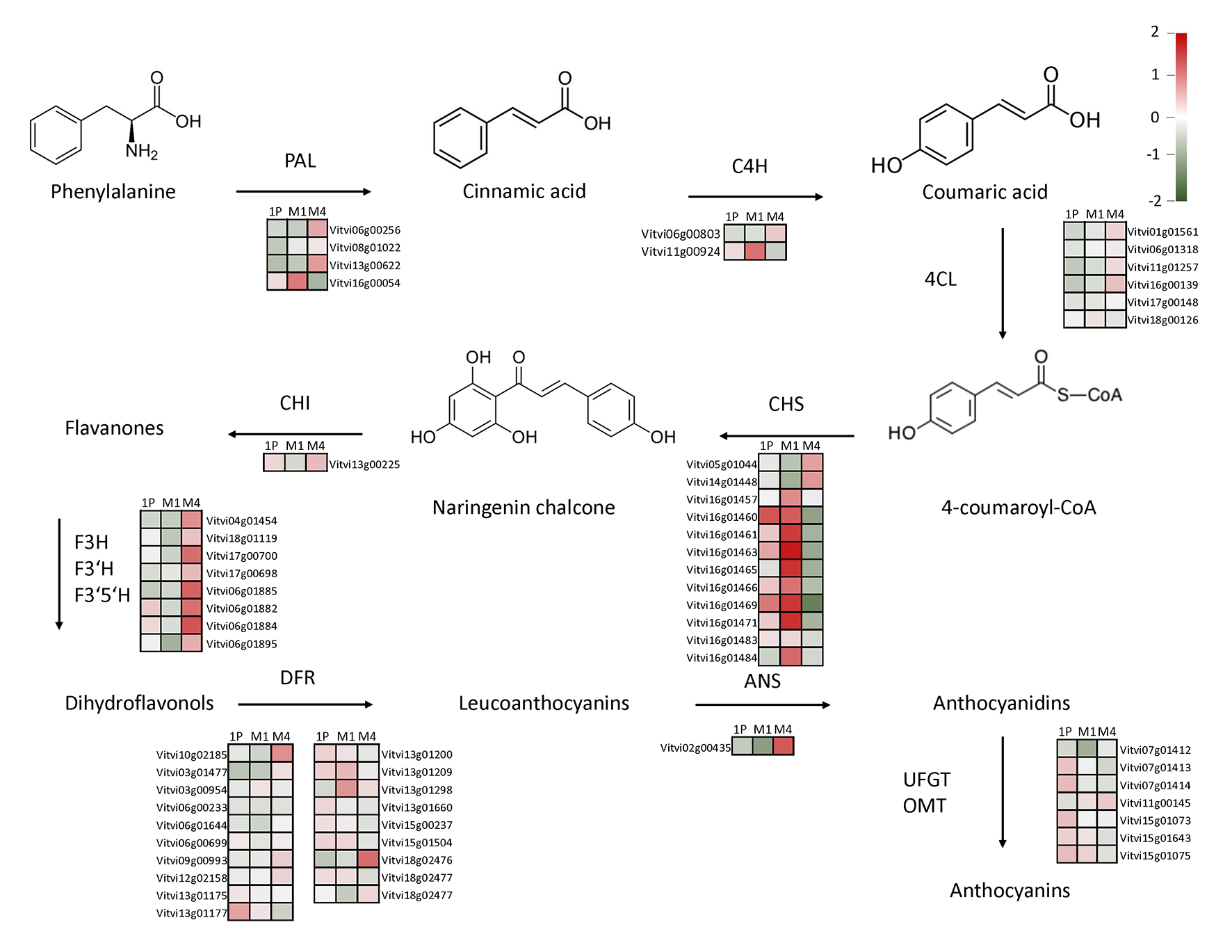

Supplement: Supplementary Figure 2 — Gene expression overview in leaf samples. (A) Hierarchical clustering analysis of the 500 most variable genes in leaf samples. (B) Principal component analysis of the berry skin samples. PC, principal component; 1P, 1103-Paulsen; C, control; S, salinity. [file Image_2.tif]

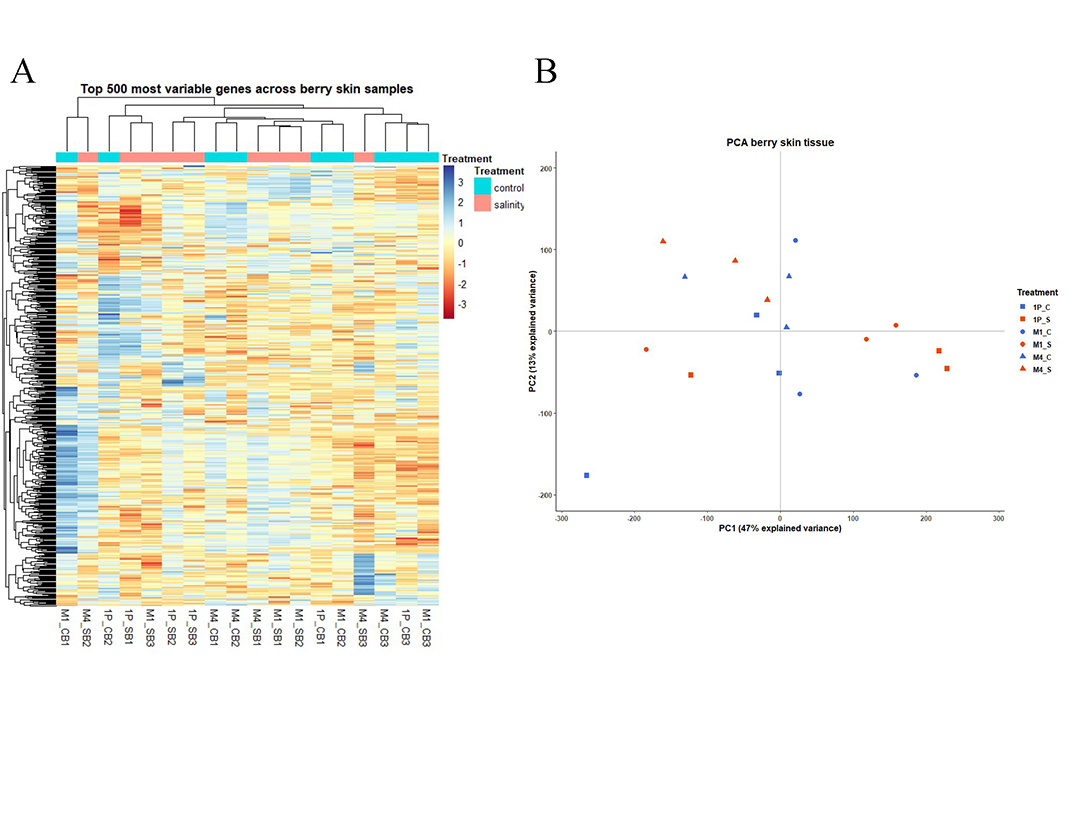

Supplement: Supplementary Figure 3 — Log2 FC values of genes involved in anthocyanin synthesis in Tempranillo in salinity-treatment leaves as compared to controls grafted onto 1103-Paulsen, M1, and M4. The specific gene names are provided by means of the Vitvi identifiers. Color represents the value of Log2 FC. PAL, phenylalanine ammonia-lyase; C4H, cinnamate-4-hydroxylase; C4L, 4-coumarate: CoA ligase; CHS, chalcone synthase; CHI, chalcone-flavanone isomerase; F3H, flavanone 3-hydroxylase; F3′H, flavonoid 3′-hydroxylase; F3′5′H, flavonoid 3′5′-hydroxylase; DFR, dihydroflavonol 4-reductase; ANS, anthocyanin synthase; UFGT, anthocyanidin 3-O-glucosyltransferase; OMT, O-methyltransferase; 1P, 1103-Paulsen rootstock; M1, M1 rootstock; M4, M4 rootstock. [file Image_3.tif]

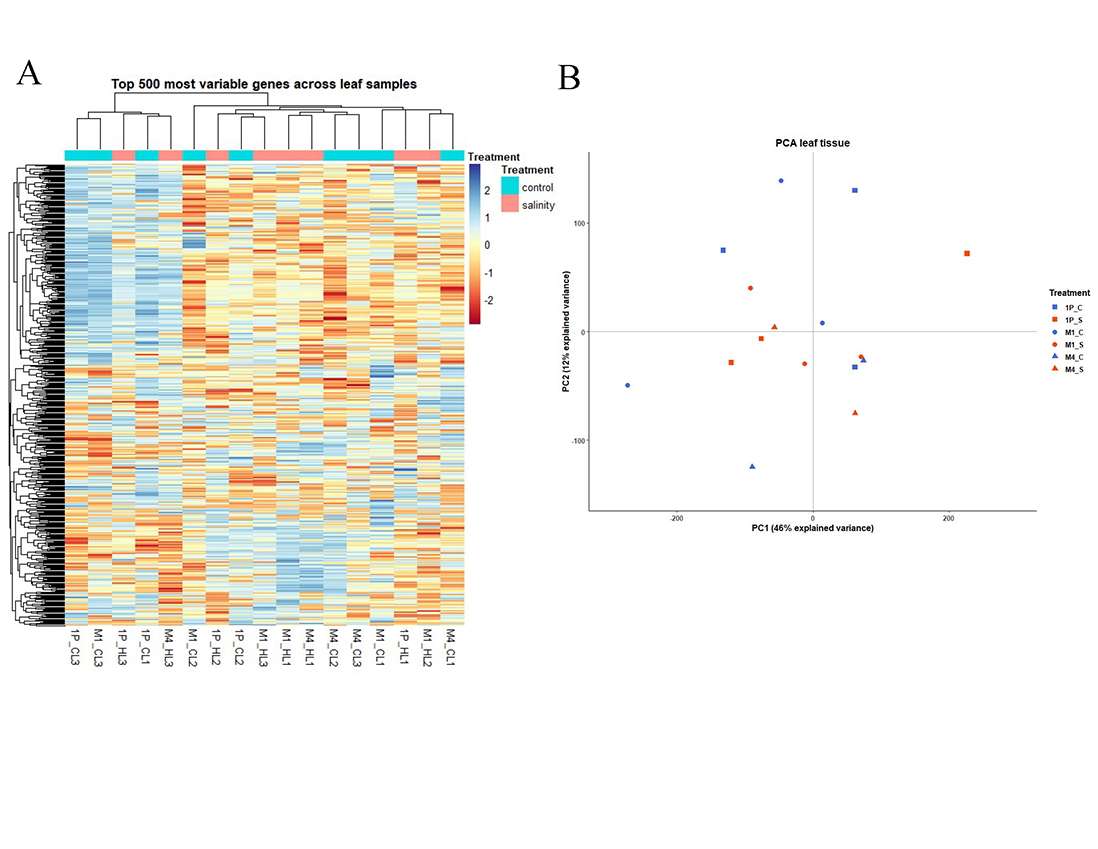

Supplement: Supplementary Figure 4 — Correlation between RNA-Seq and qPCR differential expression represented as log2 FC. R2 = 0.83. [file Image_4.tif]
